# High-speed mid-infrared laser absorption spectroscopy of CO$_2$ for shock-induced thermal non-equilibrium studies of planetary entry

**DOI:** 10.1007/s00340-022-07934-4

**Published:** 2022-11-15

**Authors:** Christopher C. Jelloian, Nicolas Q. Minesi, R. Mitchell Spearrin

**Affiliations:** Department of Mechanical and Aerospace Engineering, University of California, Los Angeles (UCLA), CA 90095 USA

## Abstract

A high-speed laser absorption technique is employed to resolve spectral transitions of CO$_2$ in the mid-infrared at MHz rates to infer non-equilibrium populations/temperatures of translation, rotation and vibration in shock-heated CO$_2$ - Ar mixtures. An interband cascade laser (DFB-ICL) resolves 4 transitions within the CO$_2$ asymmetric stretch fundamental bands ($\Delta$v$_3$ = 1) near 4.19 $\upmu \hbox {m}$. The sensor probes a wide range of rotational energies as well as two vibrational states (00$^0$0 and 01$^1$0). The sensor is demonstrated on the UCLA high enthalpy shock tube, targeting temperatures between 1250 and 3100 K and sub-atmospheric pressures (up to 0.2 atm). The sensor is sensitive to multiple temperatures over a wide range of conditions relevant to Mars entry radiation. Vibrational relaxation times are resolved and compared to existing models of thermal non-equilibrium. Select conditions highlight the shortcomings of modeling CO$_2$ non-equilibrium with a single vibrational temperature.

## Introduction

Mars and Venus planetary entries are governed by the non-equilibrium chemical kinetics (vibrational excitation, chemistry, and radiation) of shock heated CO$_2$. Flow temperatures just behind the bow shock can be in excess of 10,000 K, and around the shoulder of the vehicle, the shock-induced temperatures and pressures are significantly lower. The thermal protection system (TPS) in front of the vehicle typically experiences the highest heat loads, however the large volume of gas in the wake radiates with line of sight to the backshell and must be considered. Recent studies [1–3] have highlighted the need to investigate mid-infrared backshell radiation from vibrationally hot CO$_2$ to assess radiative heat loads that were once thought to be negligible. The most intense radiation from CO$_2$ occurs for temperatures between 2000 and 3000 K when the vibrational bands of CO$_2$ are excited and below the dissociation limit [1, 2]. In this study an optical diagnostic is developed to probe multiple vibrational and rotational states of CO$_2$ to investigate thermal non-equilibrium associated with Mars backshell radiation.

Mission planners and TPS designers simulate a wide range of planetary entries utilizing high performance computational fluid dynamics codes that consider non-equilibrium chemistry and radiation [4–7]. Validation of the rate models employed in these codes is necessary to ensure mass-efficient TPS design. When atmospheric molecules cross the bow shock of an entry vehicle, the translational and rotational energy modes excite very rapidly and equilibrate quickly. The vibrational and electronic energy modes lag behind due to higher characteristic temperature [8–10]. The finite energy transfer rates delay the approach of the mode populations to thermal equilibrium and are observed for the vibrational mode of CO$_2$ in this study, enabled by the high temporal resolution of the diagnostic. Two approaches are taken to simulate these kinds of shock-heated flows: (1) Multi-temperature models (such as [9, 11, 12]) describe the thermal state of the gas, allowing each energy mode (translation, rotation, vibration, electronic) to be described by Boltzmann population distributions at different temperatures. (2) State-to-state models (such as [13, 14]) assume no Boltzmann distributions and each energy level population is modeled with rate equations into and out of the state. The multi-temperature approach is often preferred as it significantly cuts the computational cost and often provides for a correct description of the flow. Validation of both multi-temperature and state-to-state models against experimental data is necessary to ensure accuracy across a wide range of flight conditions.

High temperature, non-equilibrium CO$_2$ flows have been experimentally studied in ground-test facilities including shock tubes and plasma torches. Klarenaar et al. [15] used Fourier-transform infrared spectroscopy (FTIR) to measure non-equilibrium temperatures of CO$_2$ and CO in a glow discharge. The FTIR technique produces quantitative results, but requires a steady or repeatable flow and cannot be easily employed in fast-evolving single-shot shock tube experiments due to the slow measurement rate. Emission diagnostics have been utilized by various groups to measure spectral radiance and infer non-equilibrium temperatures of translation, rotation and vibration. Grimaldi et al. [16] studied a recombining CO$_2$ plasma with optical emission spectroscopy between 4.1 and 5.6 $\upmu$m. They observed differences in CO$_2$ and CO rotational temperature and reported a vibrational temperature of CO$_2$. Emission diagnostics can also be employed to improve spectral simulations, see McGuire et al. [17] where several electric transition dipole models of the CO 4th positive system were compared to VUV experiments. These measurements are important, as CO emission from this band is very significant for Mars and Venus entries at high velocities on the forward heat shield [12]. Cruden et al. [11] and MacDonald et al. [18] investigated non-equilibrium CO$_2$ at entry conditions at the NASA Ames Electric Arc Shock Tube (EAST) facility. These comprehensive emission measurements were spectrally and spatially resolved for wavelengths from the infrared to the vacuum ultraviolet (VUV). Spectral radiance was fit with the NASA radiation code NEQAIR [4] to infer temperature and number densities. Additionally, the emission technique was complemented with a laser absorption sensor that probed an infrared transition of either CO or CO$_2$ (depending on the specific experiment) and from the Doppler linewidth inferred translational temperature [18]. From measured trends in temperature and number density, rate models were investigated and tuned. Measurements of rotational and vibrational temperature relied on C$_2$ Swan band emission and electronic temperature was inferred from Planck limited radiation in the VUV. Further work was conducted by Jelloian et al. [19] using a multi-laser approach to extend the laser absorption method of [18] to measure the vibrational, rotational, and translational temperatures of CO simultaneously. While the multi-temperature CO diagnostic is useful for examining radiation after dissociation, lower effective velocities and temperatures associated with backshell heating in Mars (or Venus) entry are dominated by CO$_2$ radiation and thermal non-equilibrium kinetics.

The present study involves development and demonstration of a high-speed rovibrational multi-state sensing method for CO$_2$ in the mid-wave infrared using laser absorption spectroscopy, complementing the prior analogous work focused on CO [19]. Rapid tuning of a distributed-feedback interband cascade laser (NanoPlus) in a bias-tee circuit enables sub-microsecond spectral resolution of a cluster of rovibrational lines near 4.2 $\upmu$m. The sensor is designed to investigate radiative heating rates expected on the backshell of Mars entry vehicles via quantitative investigations of shock-heated CO$_2$ between 1500 and 3000 K for comparison with multi-temperature and state-to-state models. The optical diagnostic is demonstrated for this purpose on a high-enthalpy shock tube for measurements of vibrationally excited CO$_2$ probing rotational absorption transitions in the ground and first-excited bending mode ($01^10$) of the fundamental asymmetric stretch bands centered near 4.3 $\upmu$m. The multi-line measurement is used to infer multiple temperatures and state populations at MHz rates.

## Methods and theory

### The infrared CO$_2$ spectrum

**Fig. 1:**
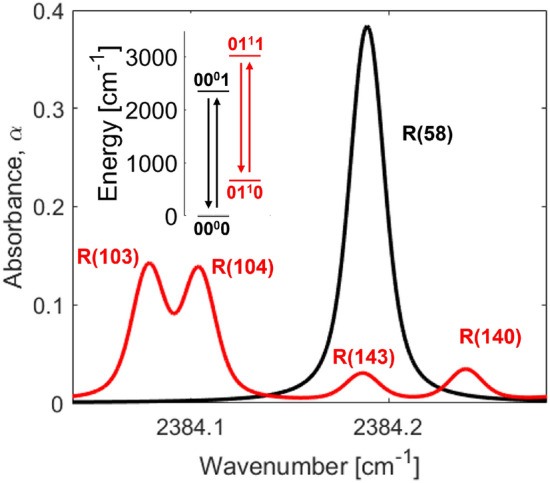
Simulated CO$_2$ spectrum using the HITEMP database [20]. The inset figure shows the energy level diagram of the vibrational states of CO$_2$ used in this study. Note the red color indicates transitions with v” = 01$^1$0.

The carbon dioxide spectrum is complicated by multiple modes of vibration with different fundamental frequencies: symmetric stretch ($\nu _1$, 1334 cm$^{-1}$), doubly degenerate bending ($\nu _2$, 667 cm$^{-1}$), and asymmetric stretch ($\nu _3$, 2349 cm$^{-1}$). Here we probe the strong absorption region near 4.3 $\upmu \hbox {m}$ which corresponds to the fundamental asymmetric stretch bands ($\nu _3$) where $\Delta$v$_3$ = 1. The fundamental asymmetric stretch bands can be distinguished by lower vibrational level, denoted with vibrational quantum numbers v$_1$v$_2^{l_2}$v$_3$ respectively, where $l_2$ characterizes the angular momentum of the molecule. Within the vibrational bands, rotational lines are indicated X(J”) where X is the branch (R, P, or Q) describing an increase, decrease, or no change in rotational quantum number, with J” being the lower state rotational assignment. The observed line intensities for absorption spectroscopy have a strong dependence on the population of the lower energy state. In this work, we probe two $\nu _3$ fundamental bands, notated as $\nu _3$(00$^0$0) and $\nu _3$(01$^1$0), and several rotational lines within the R branch of these bands that span from J”=58 to J”= 143. The main distinction between the two bands utilized in this study is the lower vibrational energy level of the bending mode, as both bands originate from the ground vibrational state of the symmetric (v$_1$ = 0) and asymmetric (v$_3$ = 0) stretch. The target absorption transitions are shown in Fig. 1 (Table 1).

**Table 1:** Rotational and vibrational lower state energies of transitions probed in this work

Line Label	$E^{''}_{\text{vib}}$	$E^{''}_{\text {rot}}$	$E^{''}_{\text {total}}$
$\nu _3(01^10)$ R(103)	667	4169	4836
$\nu _3(01^10)$ R(104)	667	4257	4924
$\nu _3(00^00)$ R(58)	0	1334	1334
$\nu _3(01^10)$ R(140)	667	7671	8338

$E^{''}_{\text {total}} = E^{''}_{\text {vib}} + E^{''}_{\text {rot}}$. Energies are given in wavenumber [cm$^{-1}$]

### Laser absorption spectroscopy

In this work, laser absorption spectroscopy (LAS) is utilized to measure the R(58) line of the $\nu _3$($00^00$) fundamental band and the R(103), R(104), and R(140) lines of the $\nu _3$($01^10$) fundamental band of CO$_2$ via spectrally-resolved light attenuation in the mid-wave infrared. Although LAS theory is well-detailed in literature [21] and has been utilized to probe rovibrational non-equilibrium of NO and CO in other studies [19, 22], key governing equations are described here for context and nomenclature definition. The Beer-Lambert law, given in Eq. 1, relates the spectral absorbance $\alpha$ at frequency $\nu$ to thermophysical gas properties via incident and transmitted light intensities, $I_0$ and $I_t$, respectively.

1 $$\begin{aligned} \alpha (\nu ) = -\ln \left( \frac{I_t}{I_0}\right) _\nu = S_j(T_{\text {rot}},T_{\text {vib}})n_AL\phi _j(\nu , T_\text {tr}, P, X_A) \end{aligned}$$

Here, $n_A$ [molec/cm$^{3}$] is the number density of absorbing molecule A, *L* [cm] is the pathlength, $S_j$ [cm$^{-1}$/(molec cm$^{-2}$)] is the linestrength of rovibrational transition *j* at rotational temperature $T_{\text {rot}}$ [K] and vibrational temperature $T_{\text {vib}}$ [K], and $\phi _j$ [cm] is the lineshape function.

In this study, scanned-wavelength direct absorption spectroscopy is utilized to resolve $\phi _j$, which is modeled using the Voigt lineshape profile. The Voigt lineshape is a convolution of Lorentzian and Gaussian profiles, capturing the effects of collisional and Doppler broadening, respectively. The Doppler broadening (Eq. 2) is used to infer the translational temperature $T_\text {tr}$ [K], where *M* [g/mol] is the molecular weight of the absorbing molecule and $\nu _{0}$ [cm$^{-1}$] is the linecenter of the transition.

2 $$\begin{aligned} \Delta \nu _D = \nu _{0}(7.1623\times 10^{-7})\sqrt{\frac{T_\text {tr}}{M}}. \end{aligned}$$

Collisional linewidth scales with broadening coefficient $\gamma _{A-B}$ [cm$^{ -1}$/atm], and partial pressure of collision partner P$_B$, as shown in Eq. 3.

3 $$\begin{aligned} \Delta \nu _C = \sum _{B}P_B2\gamma _{A-B}. \end{aligned}$$

The broadening coefficient is a function of the reduced mass of the collision partners, temperature, and collision cross-section (which is also known to be a weak function of temperature). The calculation of broadening coefficient at elevated temperatures $\gamma _{A-B}(T_{\text {tr}})$, is typically modeled via a power law fit to experimental data, as shown in Eq. 4, where $T_0$ [K] is a reference temperature and *n* is the temperature exponent.

4 $$\begin{aligned} \gamma _{A-B}(T_\text {tr}) = \gamma _{A-B}(T_0)\left( \frac{T_0}{T_\text {Tr}}\right) ^n. \end{aligned}$$

At the temperature and pressure conditions of the this study ($\Delta \nu _D$/$\Delta \nu _C$ $\sim$ 2.0), an accurate lineshape model is required to determine $T_{\text {tr}}$. The collisional broadening and temperature coefficients for CO$_2$-CO$_2$ and CO$_2$-Ar from Rosenmann et al. [23] and Lee et al. [24] were utilized as they were determined for similar rotational quantum numbers and in a similar temperature range as the present work.

To infer mode specific temperatures of rotation and vibration, the linestrength information must be accessed through the measured absorbance areas. Integration of the Beer-Lambert law (Eq. 1) over frequency yields Eq. 5 and shows the absorbance area of transition *j*, $\mathcal {A}_j$ [cm$^{-1}$], is a function of the linestrength, number density, and pathlength.

5 $$\begin{aligned} \mathcal {A}_j = S_j(T_\text {rot}, T_\text {vib})n_AL. \end{aligned}$$

The ratio of two absorbance areas is solely a function of the linestrengths as shown by Eq. 6 and a general expression for the linestrength is given in Eq. 7.

6 $$\begin{aligned} R = \frac{\mathcal {A}_1}{\mathcal {A}_2} = \frac{S_{1}(T_\text {rot}, T_\text {vib})}{S_{2}(T_\text {rot}, T_\text {vib})}, \end{aligned}$$

7 $$\begin{aligned} S_{j} = \left( \frac{N_1}{N}B_{12} - \frac{N_2}{N}B_{21}\right) \frac{h\nu }{c}. \end{aligned}$$

$B_{12}$ and $B_{21}$ [cm$^3$/(erg$\cdot$s$^2$)] are the Einstein coefficients of absorption and stimulated emission, which are calculated from the Einstein A coefficient, $A_{21}$ [s$^{-1}$], listed in the HITEMP database [20], *h* is Planck’s constant, $\nu$ is the wavenumber of the transition, *c* is the speed of light, $N_1$/N and $N_2$/N are the population fractions of the lower and upper energy states, respectively. Equation 8 can be derived from Eq. 7 assuming separable populations of rotation and vibration and Boltzmann population distributions

8 $$\begin{aligned}S_{j}& = \frac{A_{21}g_{2}}{8\pi \nu ^2cQ_\text {rot}(T_\text {rot})Q_\text {vib}(T_\text {vib})} \nonumber \\&\quad \biggl [\exp \left( \frac{-c_2E_{\text {rot},1}}{T_\text {rot}}\right) \exp \left( \frac{-c_2E_{\text {vib},1}}{T_\text {vib}}\right) \nonumber \\ &\quad -\exp \left( \frac{-c_2E_{\text {rot},2}}{T_\text {rot}}\right) \exp \left( \frac{-c_2E_{\text {vib},2}}{T_\text {vib}}\right) \biggr ], \end{aligned}$$

where $g_2$ is the upper level degeneracy of the transition, c$_2$ is the second radiation constant (1.439 cm$\cdot$K), $E_{\text {rot},1}$ and $E_{\text {vib},1}$ are the rotational and vibrational energies of the lower state [cm$^{-1}$], $E_{\text {rot},2}$ and $E_{\text {vib},2}$ are the rotational and vibrational energies of the upper state [cm$^{-1}$], and $Q_\text {rot}$ and $Q_\text {vib}$ are the partition functions of rotation and vibration. To evaluate this equation, the rotational and vibrational energies are taken from Klarenaar et al. [15], and found to be in good agreement with the values listed in HITEMP [20] and calculated from Tashkun [25]. The partition functions of NEQAIR [26] were used in this study.

The measurement sensitivity to rotational and vibrational temperatures is primarily determined from the lower state energies and the magnitude of the absorbance signals of the transitions probed. It is advantageous to probe states sufficiently separated in rotational quantum number for a sensitive rotational temperature measurement, and similarly it is advantageous to probe states sufficiently separated in vibrational quantum number for a sensitive vibrational temperature measurement. Lastly, an alternative way to determine the vibrational temperature is from the magnitude of the absorbance area signals via Eq. 5 provided the other variables are known or modeled accurately. The approach utilizing Eq. 5 is used in this study to determine $T_{\text {vib}}$ for the purpose of enhanced sensitivity.

**Fig. 2:**
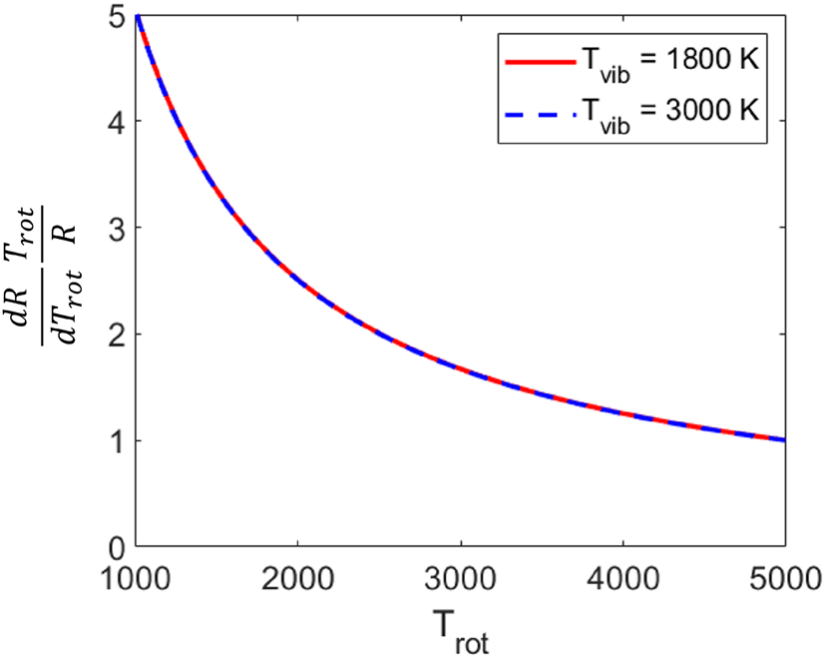
Rotational temperature sensitivity of the area ratio of the $\nu _3$(01$^1$0) R(103) and R(140) spectral features used in this study

**Fig. 3:**
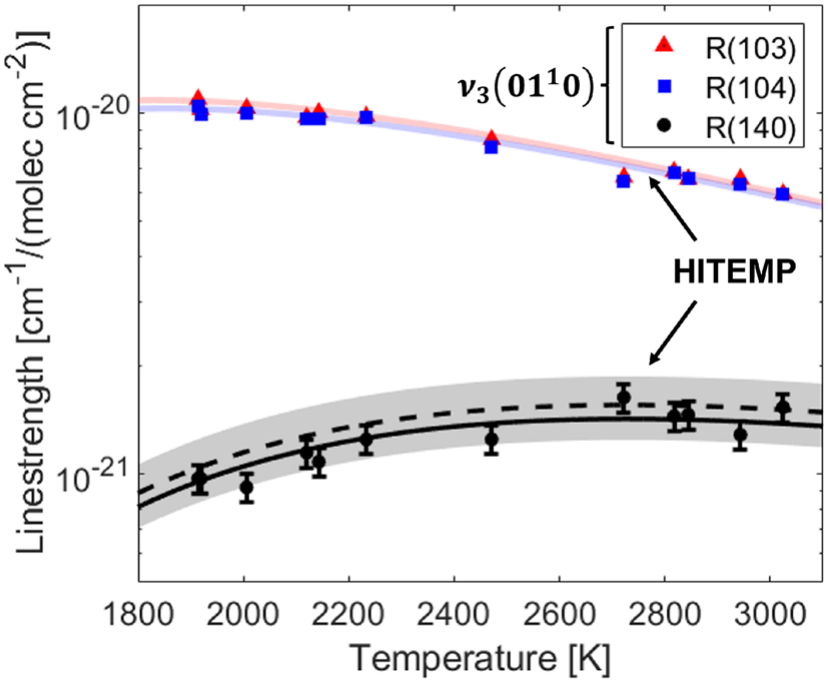
Measured equilibrium linestrength of the $\nu _3$(01$^1$0) R(103), R(104) and R(140) spectral features compared to the HITEMP model (dashed line and shaded regions) [20] over the temperature range of interest in this study. The solid line indicates the linestrength model used in this study to determine T$_{\text {rot}}$

**Fig. 4:**
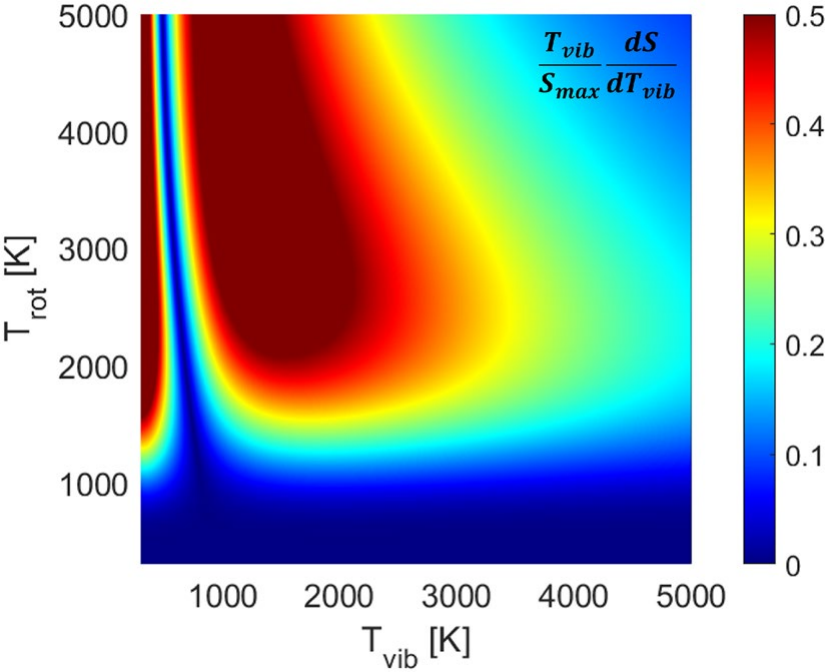
Vibrational temperature sensitivity versus rotational and vibrational temperature based on the $\nu _3$(01$^1$0) R(103) linestrength. This represents the T$_{\text {vib}}$ sensitivity based on an area magnitude change of the R(103) feature. Utilizing this method to determine T$_{\text {vib}}$ requires number density of CO$_2$ to be known

### Line selection

The sensor was designed to probe multiple rotational and vibrational state populations of CO$_2$ between 2000 and 3000 K. The wavelength region of 4.19 $\upmu \hbox {m}$ (as presented in Fig. 1) was selected for multiple reasons including: (1) the ease of resolving a clear baseline. Above 1000 K, the CO$_2$ spectrum becomes increasingly blended toward the band center near 4.3 $\upmu \hbox {m}$ and this can increase the uncertainty in resolved absorbance areas; (2) Transitions from two different vibrational states are probed (as highlighted in Fig. 1), which is not practical at shorter wavelengths beyond the $\nu _3$(01$^1$0) bandhead; (3) The targeted spectral features are sufficiently isolated to resolve the absorbance areas of several individual transitions in a relatively small domain; (4) A wide range of rotational lower state energies are probed rendering the measurement sensitive to small changes in $T_{\text {rot}}$. This is highlighted in Fig. 2 with the non-dimensional rotational temperature sensitivity of the R(103) and R(140) features plotted against $T_{\text {rot}}$ at two different vibrational temperatures. *R* is the absorbance area ratio of R(103) and R(140) shown in Eq. 6. A value greater than 1 generally indicates a sensitive measurement [27]. As noted, because the R(103) and R(140) features are from the same vibrational band ($01^10$), the area ratio is insensitive to changes in $T_{\text {vib}}$.

**Fig. 5:**
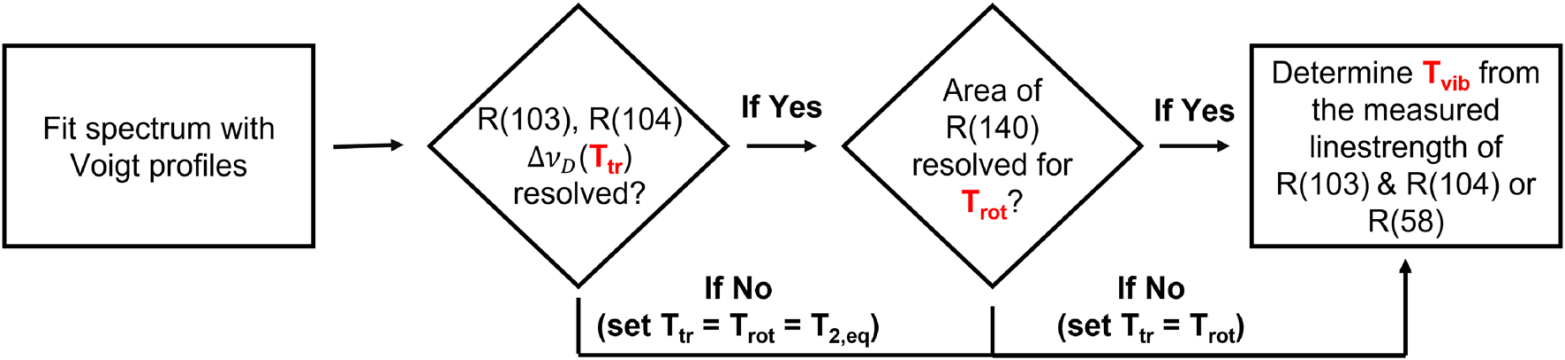
Solution method box diagram. Voigt profiles are fit to the spectrum to determine the integrated absorbance area and Doppler width

To determine $T_{\text {rot}}$, the R(140) absorbance area must be separated from the R(58) absorbance area. This is achieved via sequential fitting where the initial fit of the R(58) feature is subtracted out of the spectrum and the R(140) is fit allowing a quadratic polynomial to capture any residual curvature of the baseline. To check the efficacy of this method at known conditions, shock tube experiments were run to produce equilibrium non-reacting conditions and infer line strengths of the target CO$_2$ transitions, shown in Fig. 3. The measured values were compared to calculated linestrengths from HITEMP [20]. The measurements in this study are within the uncertainty values listed in HITEMP (1–2% for the R(103) and R(104) features, and $\ge$ 20% for the R(140) feature). On average, the R(140) linestrengh was measured to be 8.5% below the HITEMP value which is within the tabulated uncertainty, thus an adjustment of 8.5% was applied for data processing. This is highlighted by the solid line in Fig. 3.

The vibrational temperature is determined once $T_{\text {rot}}$ is known via numerically solving Eq. 5 and comparing to the measured linestrength determined from the absorbance area, $\mathcal {A}_j$. Critically, this is only feasible when mole fraction is known, as is generally expected for conditions < 3000 K, thus rendering the line intensity a function of multiple temperatures. Figure 5 illustrates the solution method. The density profile behind the incident shock is modeled by an exponential increase from the vibrationally frozen density predicted from the normal shock relations solver [28] to the equilibrium number density after relaxation. Figure 4 shows the absorbance area of the R(103) feature is most sensitive to $T_{\text {vib}}$ over the temperature range of 1500–3000 K. While any line area may be theoretically used to infer a $T_{\text {vib}}$ value, the R(103) and R(104) lines are preferentially used here as R(58) can be optically thick, and R(140) is often optically thin. It should be noted for temperatures > 3000 K, dissociation starts to affect the absorbance area and the uncertainty of $T_{\text {vib}}$ increases significantly unless the number density of CO$_2$ can be determined in an alternate way. Addressing this limitation is less critical given that at temperatures instigating dissociation, vibrational relaxation is very short, meaning that state populations approach a single temperature distribution (i.e. thermal equilibrium).

Lastly, the population fraction of the rovibrational state *j*, $N_j/N$ where *N* is the total CO$_2$ population, can be derived from Eqs. 5 and 7 using the absorbance area, $\mathcal {A}_j$, as shown in Eq. 9, provided the stimulated emission ($N_2/N$) can be estimated.

9 $$\begin{aligned} \frac{N_j}{N} = \frac{g_j}{g_2}\left( \frac{8\pi \nu ^2c\mathcal {A}_j}{A_{21}NL} + \frac{N_2}{N}\right) . \end{aligned}$$

## Optical and experimental setup

**Fig. 6:**
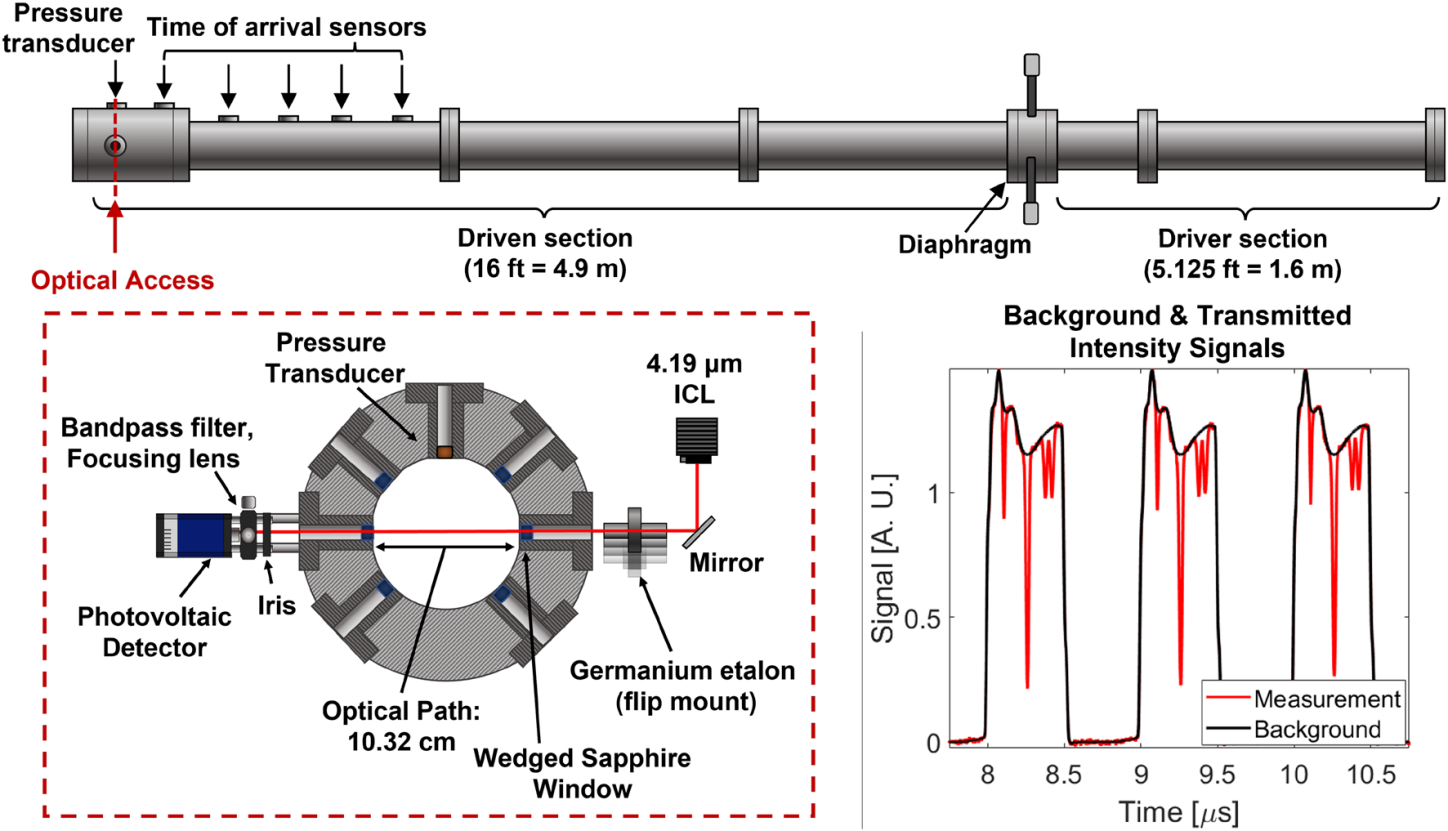
(top) Shock tube schematic. (left) Sensor layout through the optical access location 2 cm from the endwall. (right) Background and transmitted light intensity signals during an incident shock. Note the transient intensity in the measurement signal

The optical and experimental setup is shown in Fig. 6. An interband cascade laser is used to resolve four rovibrational lines of the asymmetric stretch fundamental bands of CO$_2$ near 4.19$\upmu \hbox {m}$ described above in Sec. 2.3. The laser is controlled using an RF-diplexer (bias-tee) circuit as described in [29]. A square waveform is used, as this type of modulation maximizes the signal intensity, and hence optimizes the signal to noise ratio (SNR) during the scan. An additional benefit of the square waveform includes the sharp temperature difference the laser chip experiences between the up and down scans. This temperature difference is what drives the wavelength change during the scan, and therefore the square wave maximizes the scan-depth (the range of wavelengths generated) for a given amplitude of current modulation. Additional details on improving the scan-depth using arbitrary waveforms are given in Nair et al. [30].

The sensor has a relatively simple optical setup as shown in Fig. 6. Key hardware and sensor operation is briefly discussed here. It can be seen in the sample laser scans of Fig. 6 that the laser is scanned below the lasing threshold. This allows for an accurate accounting of the emission during each scan, and the detector dark signal to be known immediately before the test. The total integration time of one scan is 500 ns, as the laser is effectively off (not emitting) when the current is below the lasing threshold limit. This scan function yields an effective measurement rate of 1 MHz. A 2-inch germanium etalon is used to convert light intensity signals from the time domain to the wavenumber domain. The background intensity and etalon signals are recorded immediately before each run. Additional hardware include a narrow bandpass filter and iris to mitigate the emission signal prior to light detection. Two wedged sapphire windows were used to seal the shock tube and prevent etalon signals between the two windows, and a focusing lens was used to collect the light onto a high bandwidth ($\sim$200 MHz) photovoltaic detector and mitigate beam steering. Additionally, beam steering tests were performed throughout the test series to ensure accurate measurements immediately behind the passage of the incident shock. Lastly, the data acquisition system has a bandwidth of 200 MHz and recorded signals at 12-bits and a sampling rate of 1.25 GS/s providing high-SNR measurements of the raw light intensities.

A high enthalpy shock tube (HEST) at UCLA was used for all experiments in this work to shock-heat CO$_2$ - Ar over a range of incident shock velocities up to 2.7 km/s relevant to Mars entry backshell heating. Different mixtures of CO$_2$ - Ar were used with CO$_2$ concentrations of 2$\%$, 20$\%$, and 100$\%$. The facility is well documented in literature [31] and briefly described here. The stainless-steel tube consists of a 5.125 ft cold gas driver and a 16 ft driven section with 5 piezoelectric transducers integrated along the length of the driven section to measure the shock position/time of arrival and infer shock velocity. A high-speed pressure transducer (Kistler 601B1) and optical access are located 2 cm from the endwall. Before each test, a turbopump is used to achieve an ultimate vacuum pressure on the order of 100 s of $\upmu \hbox {torr}$ and leak rates are measured via rate of rise on a Baratron manometer (627D) and were in the range 0.1–1 mtorr/min.

## Results/sensor demonstration

Based on the sensing strategy and experimental setup outlined in the previous sections, a series of tests were performed to validate the sensor at controlled conditions. Additionally, a number of tests were conducted to examine thermal non-equilibrium of CO$_2$ and associated vibrational relaxation timescales, with comparison to existing models.

**Fig. 7:**
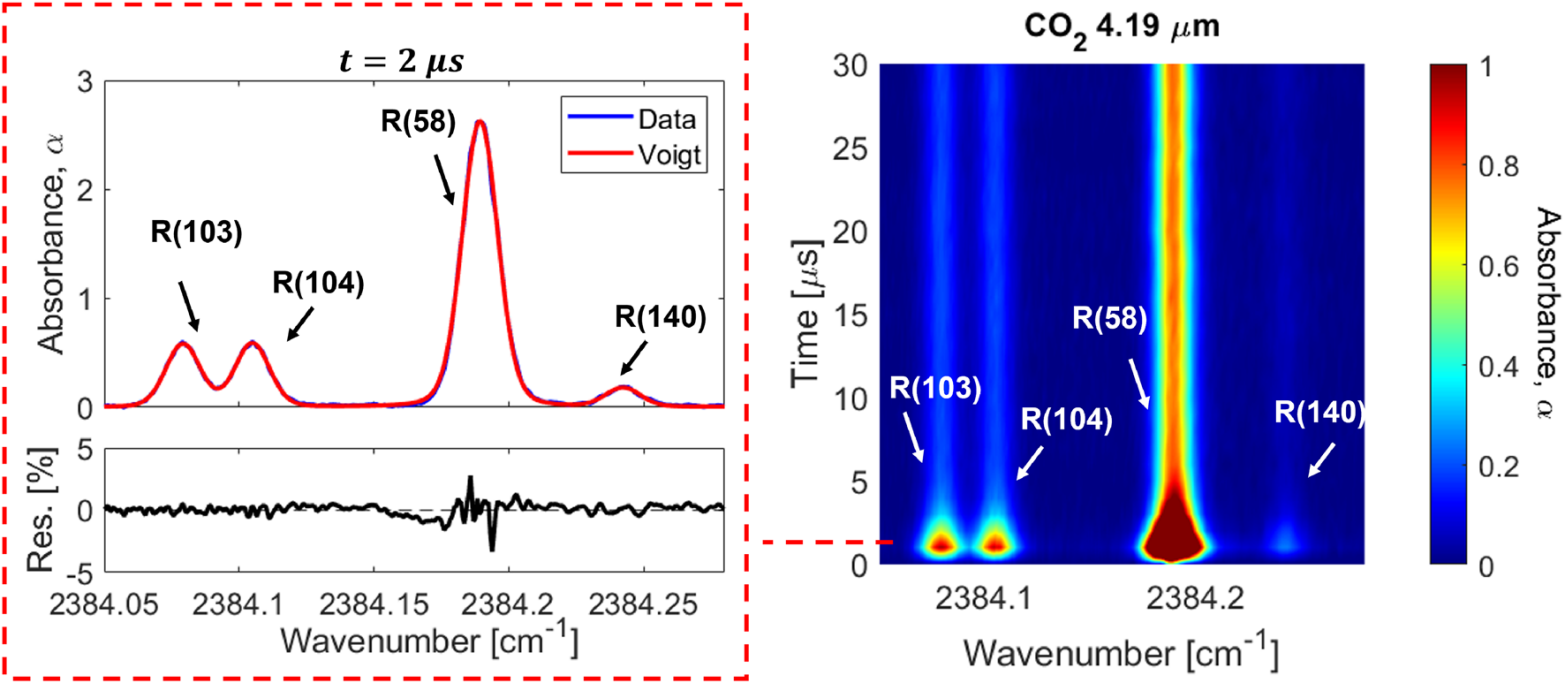
(left) Voigt Fit of the spectra at t = 2 $\mu$s into the test. (right) 2D map of absorbance versus time and wavenumber. Shock conditions: Fill pressure = 1.30 Torr, 20% CO$_2$ - Ar, U$_{\text {is}}$ = 1855 ± 15 m/s

### Multi-temperature validation

Incident shock waves with velocities between 1.1 and 2.7 km/s in CO$_2$ - Ar gas mixtures were studied to determine the range, accuracy and precision of the sensor with regards to translational, rotational, and vibrational temperature measurements. Equilibrium temperatures were achieved between 1250–3100 K and equilibrium pressures ranged from 0.03 to 0.17 atm. These conditions prevented any CO$_2$ dissociation during the test time.

**Fig. 8:**
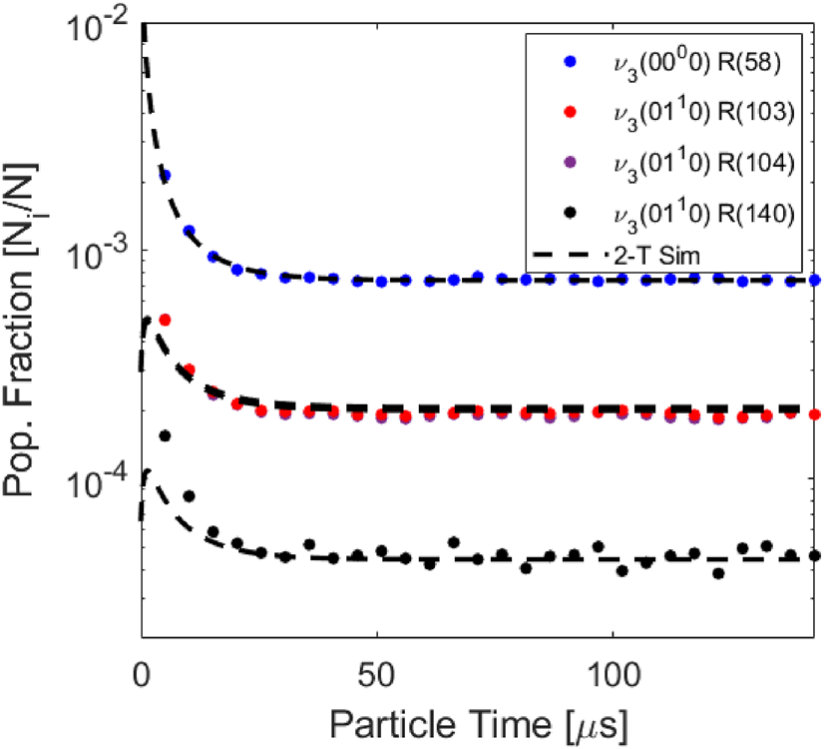
Measured populations versus time compared to the two temperature Simpson relaxation time model [32] from the normal shock relations solver [28]. Shock conditions: Fill pressure = 1.30 Torr, 20% CO$_2$ - Ar, U$_{is}$ = 1855 ± 15 m/s

As described in Sect. 2, the temperatures of rotation and vibration are related to the population distribution of CO$_2$ and determined from the absorbance area measurements of the R(58), R(103), R(104), and R(140) rovibrational lines in the $\nu _3$ bands. Figure 7 (right) shows a representative absorbance signal resolved versus wavenumber and time. A clear intensity reduction occurs in the first $5\upmu \text {s}$ of the test, related to a population decrease in the v”= 0$0^0$0 and v”= 0$1^1$0 energy levels. The population fractions are plotted versus time using Eq. 9 and compared with the 2-temperature vibrational relaxation model of Simpson et al. [32] in Fig. 8. The observed decrease in population fraction of these states is expected because the ground and first bending mode are low vibrational states (E$_{\text {vib}, 01^10}$ = 667 cm$^{-1}$). Therefore, they will depopulate during vibrational excitation at temperatures greater than approximately 1000 K. After the initial transient, strong agreement is observed with the simulated equilibrium population fraction. At this condition, good agreement is generally observed between the values predicted from Simpson et al. [32] throughout the entire test.

The time axis in the following figures is given in particle time as this facilitates easier comparisons to relaxation rates given in literature and is related to the lab frame via Eq. 10 [33].

10 $$\begin{aligned} \Delta t_{\text{particle}} = \frac{\rho _2}{\rho _1}\Delta t_{\text {lab}}. \end{aligned}$$

The relationship between particle time ($\Delta t_{\text {particle}}$) and lab time ($\Delta t_{\text {lab}}$) in Eq. 10 can be derived from ideal shock theory by relating the distance travelled by the shock and the distance travelled by a particle in the flow assuming conservation of mass across the shock wave. Particle time must be taken into account in the study of incident shock waves as the passage of the incident shock induces a velocity on the particles.

**Fig. 9:**
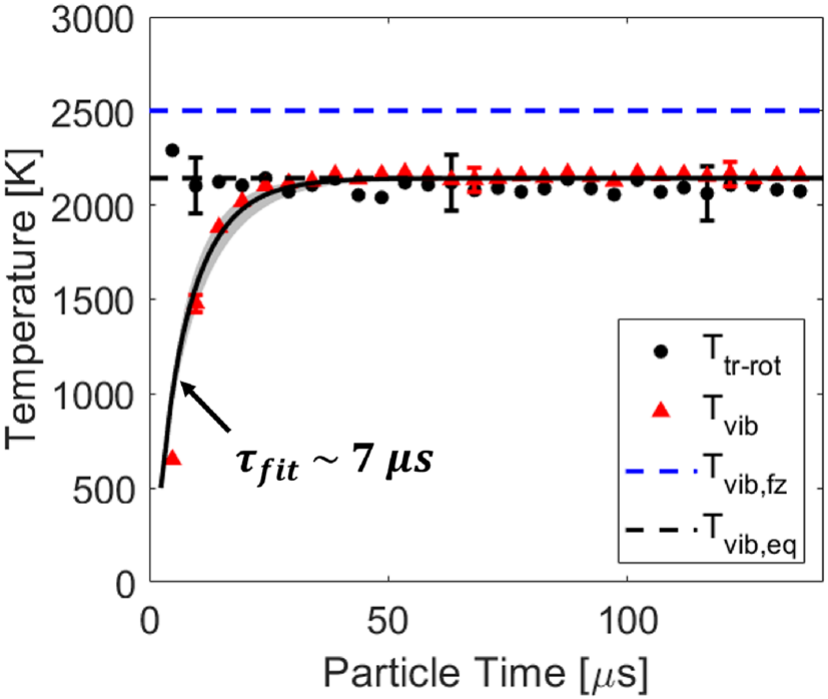
Time resolved temperatures compared to the normal shock relations solver [28]. Shock conditions: fill pressure = 3.50 Torr, 20% CO$_2$/Ar, U$_{\text {is}}$ = 1612 ± 11 m/s

**Fig. 10:**
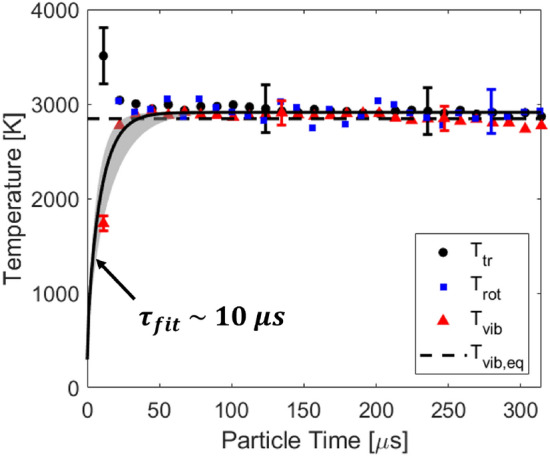
Time resolved temperatures compared to the normal shock relations solver [28]. Shock conditions: fill pressure = 0.36 Torr, 100% CO$_2$, U$_{\text {is}}$ = 2562 ± 24 m/s. Note the vibrationally frozen temperature is estimated at 5100 ± 94 K and not shown on the figure

Time resolved temperatures determined using the process outlined in Fig. 5 are presented in Figs. 9 and 10 for temperatures near 2100 K and 3000 K, respectively. A clear energy transfer is observed behind the incident shock passage at *t* = 0 from the translational and rotational energy modes to the vibrational energy mode. This relaxation is due to both vibration-vibration (VV) and vibration-translation (VT) exchange processes in the gas. For the case presented in Fig. 10, good agreement is seen between the translational and rotational energy modes of the gas. This is expected because translational and rotational energy modes equilibrate significantly faster than the vibrational energy mode [14, 34]. The energy modes are also seen to equilibrate to the temperature predicted from the normal shock relations solver [28], indicating an accurate and quantitative temperature measurement.

**Fig. 11:**
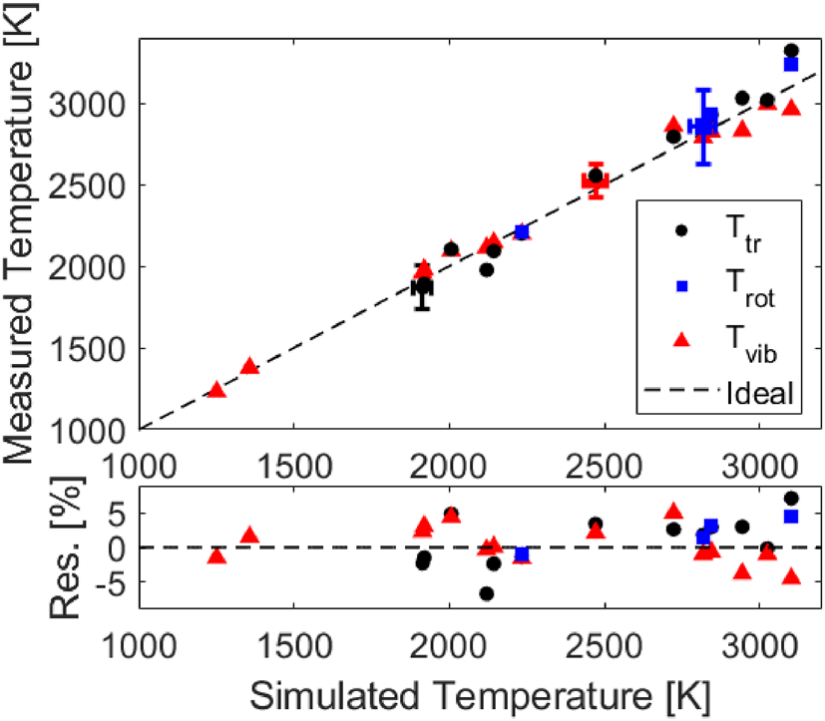
Mean equilibrium temperatures of translation, rotation, and vibration compared to the normal shock relations solver [28]. Temperature ranges from 1250–3100 K

Further tests were conducted to validate the sensor over a range of temperatures of interest (1250–3100 K) and assess its limitations. Measured equilibrium temperatures are compared to the normal shock relations solver [28] and results are presented in Fig. 11. Representative error bars are calculated using the method described in Sect. 6 largely following the analysis described in [29, 35]. In this figure, the representative error bars are estimated as 7% for $T_{\text {tr}}$, 4% for $T_{\text {vib}}$, and 8% for $T_{\text {rot}}$. Note rotational temperatures are reported on 100% CO$_2$ cases where the R(140) feature can be sufficiently resolved. Below $\sim$2200 K, where the R(140) can not be well-resolved, the rotational and translational temperatures can not be determined independently and are thus assumed to be equal. The primary domain of operation for multi-temperature sensing is 1900–3100 K. In more dilute gas mixtures wherein translational and rotational temperatures are equilibrated immediately behind the shock and determined by normal shock relations, the vibrational temperature may still be measured independently, demonstrated here down to 1250 K. The average discrepancy between measured temperatures in the equilibrium region with calculated temperatures from normal shock relations is $\sim$4%. The average standard deviation (2$\sigma$) of the measured temperatures after vibrational relaxation is found to be 105 K for $T_\text {tr}$, 164 K for $T_\text {rot}$ and 106 K for $T_\text {vib}$, on the order of 5% of the measurement values, representing measurement precision.

### Vibrational relaxation timescales

From resolved vibrational temperature trends, vibrational relaxation time is determined and compared to the models of Simpson [32] and Park [9]. The Simpson model was developed from a laser Schlieren technique which measured the density change during vibrational relaxation behind incident shock waves in CO$_2$ - Ar mixtures. The Park model was fit to the pure CO$_2$ data of Carmac [36] and relies on the correlation formula of Millikan and White [37] to estimate the vibrational relaxation rate with Ar. The functional form of each model is subtly different, with Park adding an additional term to prevent the vibrational excitation rate from exceeding the elastic collision cross section ($\sigma '$). The value Park lists for $\sigma '$ has negligible effect at the conditions of this study.

The fitting procedure used to determine $\tau _{vib}$ in this work follows from the Bethe-Teller equation shown in Eq. 11.

11 $$\begin{aligned} {\frac{\text {d}e_\text {vib}}{\text {d}t}} = {\frac{e_\text {vib}^*(T_\text {tr}) - e_\text {vib}(T_\text {vib})}{\tau _{\text {vib}}}}, \end{aligned}$$

e$_\text {vib}^*$ is the vibrational energy the gas would have if it were at the translational temperature of the gas. The vibrational energy of the gas is calculated from the measured vibrational temperature via Eq. 12 assuming three separable modes of vibration, and a simple harmonic oscillator to approximate the energy level spacing within the modes. $\Theta _{\text {vib}}$ [K] is the characteristic vibrational temperature of the mode and k is the Boltzmann constant. The solution of the Bethe-Teller equation for a bath gas is given in Eq. 13, and is linearly fit on a log plot to determine $\tau$ from the slope. e$_{\text {vib}}$(T$_{\text {vib},i}$) is the initial vibrational energy of the gas before relaxation.

12 $$\begin{aligned} e_{\text {vib}} = \frac{n_Ak\Theta _{\text {vib}}}{\exp \left(\frac{\Theta _{\text {vib}}}{T_{\text {vib}}}\right)-1}, \end{aligned}$$

13 $$\begin{aligned} \frac{e_{\text {vib}}^*(T_{\text {eq}})-e_{\text {vib}}}{e_{\text {vib}}^*(T_{\text {eq}}) - e_{\text {vib}}(T_{\text {vib},i})} = \exp \left(\frac{-t}{\tau }\right). \end{aligned}$$

**Fig. 12:**
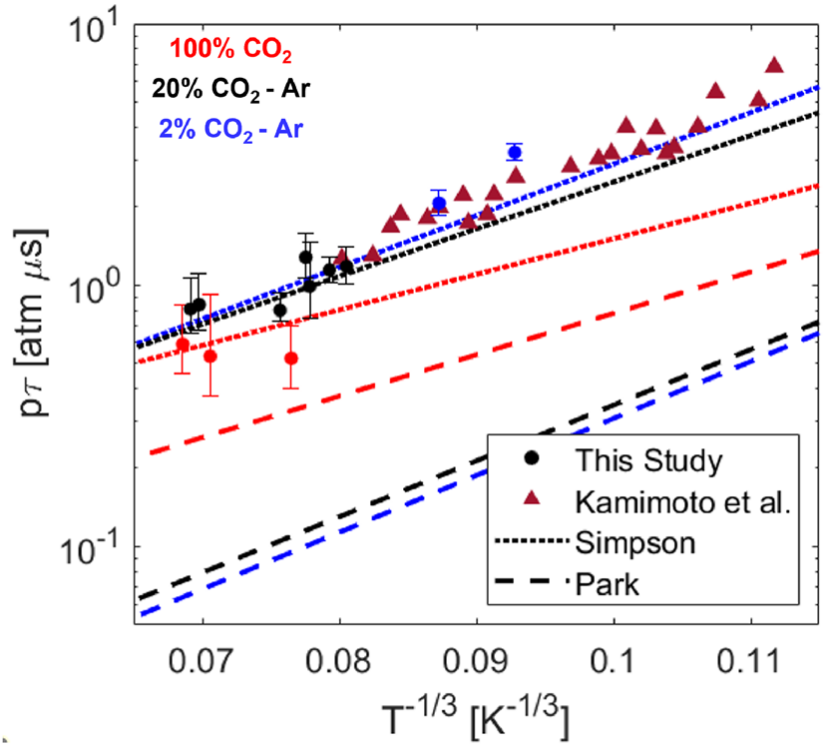
Landeau-Teller plot of relaxation times measured in this study compared to literature. Mixture concentrations are denoted with the blue (2% CO$_2$-Ar), black (20% CO$_2$-Ar), red (100% CO$_2$) color scheme

The fitted relaxation time is shown in Fig. 10 and estimated to be 7 ± 2 $\upmu$s, based on the uncertainty in the fitted slope. The vibrational relaxation times were fit over the range of conditions investigated in this study and are presented in Fig. 12. Good agreement is seen with the data measured by Kamimoto et al. [38] who studied mixtures $\le$ 4% CO$_2$ - Ar with an emission technique. For dilute CO$_2$ in argon, the Simpson model [32] was found to be largely within the uncertainty of the measurements. The Park rate for CO$_2$ - Ar is based off an empirical correlation formula developed by Millikan and White [37]. Therefore, it is expected that the experimentally determined rate of Simpson better captures the data. Additionally, the Simpson model predicts faster vibrational relaxation with increasing CO$_2$ concentration and this is consistent with the measurements of this study.

### State population analysis

**Fig. 13:**
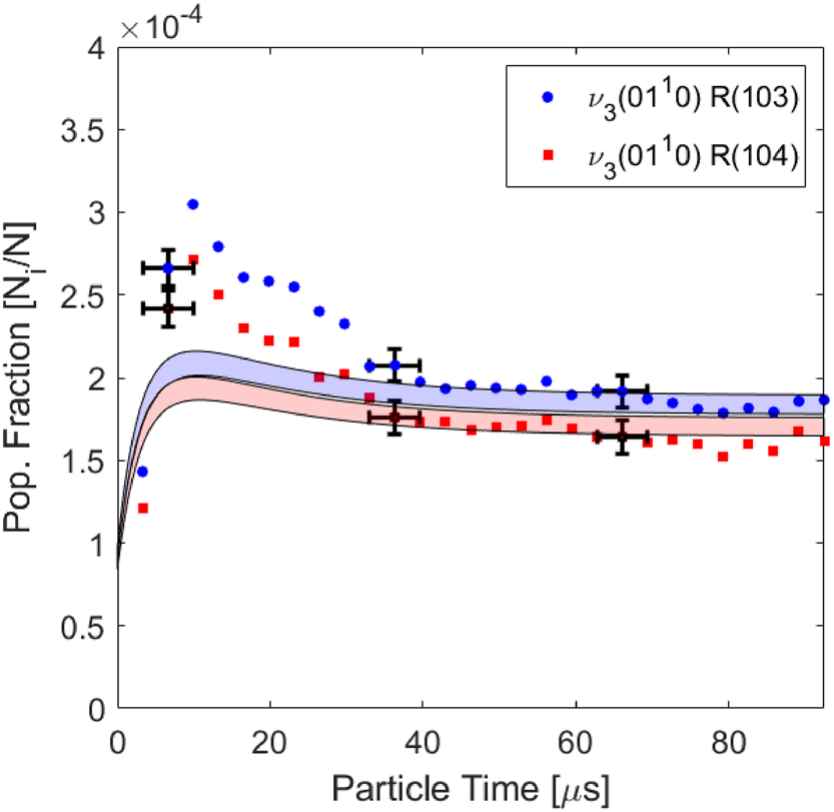
Time-resolved population fractions in an incident shock compared to a two-temperature model assuming the relaxation rate of Simpson et al. [32]. Shock conditions: Fill pressure = 5.60 Torr, 2% CO$_2$/Ar, U$_{\text {is}}$ = 1116 ± 7 m/s

In addition to multi-temperature analysis, the spectroscopic technique may be used to infer state-specific populations (per Eq. 9) in thermal non-equilibrium and inform state-to-state modeling efforts. Figure 13 demonstrates one case of particular interest at the low end of the investigated shock velocities. The measured population fractions of two rovibrational states ($01^10$, J”=103 and J”=104) are compared to the calculated ones assuming a single vibrational temperature and the Simpson relaxation time. At this test condition, and a few others at similarly weak shock velocities, the multi-temperature solution method failed to recover a $T_{\text {vib}}$ from the first several recorded spectra after the shock front. This was particularly noticeable on the lowest CO$_2$ concentration test cases where VV rates are expected to have the least effect due to the limiting of CO$_2$ - CO$_2$ collisions and also where the kinetics are expected to be the slowest at low temperatures. Since $T_{\text {vib}}$ is determined from Eq. 5, and the number density and pathlength are well known, it is suspected the two temperature linestrength model (Eq. 8) is not valid at these conditions. A clear overpopulation of the 01$^1$0 states are measured for approximately 25 $\upmu$s of particle time. To generate the model uncertainty displayed in Fig. 13 as the shaded region, the listed 2% uncertainty in the database linestrength value was considered along with the partition function uncertainty. The partition function was simulated at both the vibrationally frozen rotational temperature (1375 K) and vibrationally equilibrated rotational temperature (1357 K). These temperatures are very similar because the CO$_2$ is very dilute (2%) in Ar. It is observed the uncertainty in $T_{rot}$ and the uncertainty in the evaluation of the partition function cannot explain the gap between the measured population fraction and the two temperature model, though the timescale is well captured by the Simpson rate [32].

The likely explanation of the result displayed in Fig. 13 is non-equilibrium between the bending and asymmetric stretch vibrational modes ($T_{\text {vib,bend}}$ $\ne$ $T_{\text {vib,as}}$). It is known the asymmetric stretch temperature, $T_{\text {vib,as}}$, relaxes slower than the symmetric stretch and bending modes [34]. If $T_{\text {vib,as}}$ is lower than $T_{\text {vib,ss}}$ and $T_{\text {vib,bend}}$, the stimulated emission term in Eq. 9 is overestimated. Thus, lowering $T_{\text {vib,as}}$ would decrease the inferred population fraction of this state from the absorbance data. Simultaneously, lowering $T_{\text {vib,as}}$ would increase the expected population fraction in the level, thus bringing the measurement and the model into better agreement. Rigorously testing this hypothesis is beyond the scope of this paper, but it is clear that the MHz-rate state population measurements enabled by the sensing method provide an opportunity for such future work.

## Conclusion

A mid-IR laser absorption sensor has been developed and demonstrated to probe several rovibrational state populations of CO$_2$ at MHz rates in shock-induced thermal non-equilibrium relevant to Mars entry heating. Shock tube tests were performed to examine sensor capability with shock velocities between 1.1 and 2.7 km/s in various CO$_2$-Ar gas mixtures ranging from 2%, 20%, and 100% CO$_2$. Quantitative state populations are used to infer translational, rotational and vibrational temperatures. Pressure and equilibrium temperature conditions ranged from 0.03 to 0.17 atm and 1250 to 3100 K. This sensing method was shown to provide quantitative results for temperature across this range, with estimated uncertainties of 7% for $T_{\text {tr}}$, 4% for T$_{\text {vib}}$, and 8% for T$_{\text {rot}}$. Demonstrated temperature precision was approximately $\sim$100 K for each energy mode. The high effective precision, accuracy, and temporal resolution (1 $\upmu \hbox {s}$ in the lab frame) of this sensor demonstrates its potential for use to investigate the complex vibration - dissociation dynamics of CO$_2$.

From the tests performed in this work, thermal non-equilibrium was investigated and compared to rates of vibrational excitation predicted from the Park [9] and Simpson [32] models. Our results show good agreement with the Simpson model and the measurements of Kamimoto et al. [38]. On a few tests cases at low shock velocity, a single vibrational temperature could not be determined at early test times. This was primarily observed on low temperature tests highly dilute in Ar, likely due to the limiting of VV exchanges during CO$_2$-CO$_2$ collisions and low collision rates in this temperature range. It should be noted that the rate model of Simpson [32] still predicted the relaxation timescale well at these conditions, however the two temperature linestrength model fails in the prediction of the population distribution of the (01$^1$0) state. Non-equilibrium between the asymmetric stretch and bending vibrational modes likely explains the discrepancies between the two-temperature model and measured population fractions. Further analysis requires a model of the asymmetric stretch temperature or additional spectral information. Specifically, additional lines representing each vibrational mode should be measured for inference of multiple vibrational temperatures of CO$_2$. Additionally, it would be advantageous to probe a wider range of lower state vibrational energies to decouple the vibrational temperature measurement from the number density. In summary, a new high-speed laser absorption sensing technique has been developed to investigate CO$_2$ non-equilibrium processes at relevant temperatures and pressures to Mars backshell heating and demonstrated to yield quantitative results to refine non-equilibrium rate models. This diagnostic can be used to complement the state-of-the-art emission diagnostics utilized by various groups to reduce uncertainties in models of thermal non-equilibrium between translational, rotational, and vibrational energy modes of CO$_2$.

## Figures and Tables

**Fig. 14:**
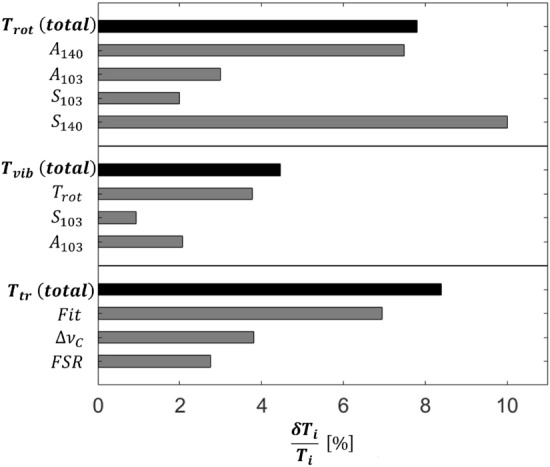
Calculated relative uncertainty contribution from each term in Eq. 19 (top), Eq. 17 (middle), and Eq. 16 (bottom) for the case presented in Fig. 10

**Fig. 15:**
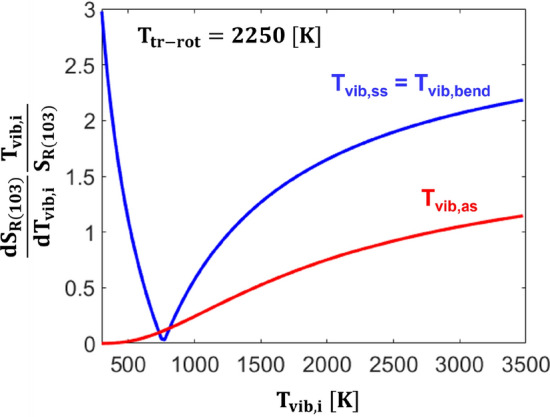
Calculated sensitivity of the $\nu _3$(01$^1$0) R(103) area to the bending mode temperature and asymmetric stretch mode temperature as a function of respective vibrational mode temperature

## Appendix

### Temperature uncertainty analysis

This section describes the calculation of uncertainty based on the Taylor series method of error propagation and assuming uncorrelated sources of uncertainty [39]. The non-dimensional uncertainty of a dependent variable r ($\delta$r/r) can be calculated from the uncertainty in the independent variables ($\delta$x$_i$) used to calculate r as shown in Eq. 14.

14 $$\begin{aligned} {\left( \frac{\delta r}{r}\right) ^2 = \sum _{i} \left( {\frac{\partial r}{\partial x_i}} {\frac{\delta x_i}{x_i}}{\frac{x_i}{r}}\right) ^2 }. \end{aligned}$$

For the uncertainty in translational temperature, uncertainty in the free spectral range (FSR) of the etalon used to make the time to wavenumber transform is considered as well as the uncertainty in the collision width and Voigt fit as shown in Eqs. 15 and 16.

15 $$\begin{aligned} {\left( \frac{\delta T_{\text {tr}}}{T_{\text {tr}}}\right) ^2 = \left( {\frac{\partial T_{\text {tr}}}{\partial \nu _D}} {\frac{\delta \nu _D}{\nu _D}}{\frac{\nu _D}{T_{\text {tr}}}}\right) ^2 }, \end{aligned}$$

16 $$\begin{aligned} {\left( \frac{\delta \nu _D}{\nu _D}\right) ^2 = \left( {\frac{\delta \nu _{D,\text {FSR}}}{\nu _D}}\right) ^2 + \left( {\frac{\delta \nu _{D,\Delta \nu _C}}{\nu _D}}\right) ^2 + \left( {\frac{\delta \nu _{D,\text {Fit}}}{\nu _D}}\right) ^2 }. \end{aligned}$$

The derivative of $T_{\text {tr}}$ with Doppler width is easily calculated from Eq. 2. The FSR uncertainty is estimated at 1% and the spectrum is re-fit with the adjusted FSR. The uncertainty in collision width for CO$_2$-CO$_2$ collisions from Rosenmann et al. [23] is listed as 7%, and CO$_2$-Ar collision width uncertainty is 3% from the values listed in Lee et al. [24]. The Voigt fit uncertainty is taken as the root mean square of the residual of the fit over the spectral features estimated as approximately 2.5%. Note that as the temperature increases and pressure decreases, the uncertainty due to the collision width will diminish, increasing the precision of the $T_{\text {tr}}$ measurement. Figure 14 (bottom) shows the relative contributions of each source of error in the Doppler width measurement. The primary sources of uncertainty arise in the fit, and in the collision width.

The uncertainty in vibrational temperature is determined from the uncertainty in absorbance area, linestrength, and rotational temperature via Eq. 17.

17 $$\begin{aligned}\left( \frac{\delta T_{\text {vib}}}{T_{\text {vib}}}\right) ^2& = \left( \frac{\partial T_{\text {vib}}}{\partial A} \frac{\delta A}{A}\frac{A}{T_{\text {vib}}}\right) ^2  \nonumber \\&\quad  +\left( {\frac{\partial T_{\text {vib}}}{\partial S}} {\frac{\delta S}{S}}{\frac{S}{T_{\text {vib}}}}\right) ^2 + \left( {\frac{\partial T_{\text {vib}}}{\partial T_{\text {rot}}}} {\frac{\delta T_{\text {rot}}}{T_{\text {rot}}}}{\frac{T_{\text {rot}}}{T_{\text {vib}}}}\right) ^2. \end{aligned}$$

The uncertainty in absorbance area *A* can be estimated via Eq. 18, where $\alpha _{pk}$ is the peak absorbance of the transition [35].

18 $$\begin{aligned} {\frac{\delta A}{A} = \frac{1}{\text {SNR}}\left( \frac{\exp (\alpha _{pk})}{\alpha _{pk}}\right) }. \end{aligned}$$

The derivative of vibrational temperature with area and linestrength is calculated numerically from Eq. 5 and Eq. 8. The derivative of $T_{\text {vib}}$ with $T_{\text {rot}}$ is determined numerically via a perturbation approach where $T_{\text {vib}}$ is recalculated after $T_{\text {rot}}$ is perturbed in the model (consistent with the estimated uncertainty of $T_{\text {rot}}$ on a given test). $\delta$S/S is estimated from the uncertainty value in the reference linestrength listed in HITRAN [40] for R(103) and R(104) (2 %) and HITEMP [20] for R(140) (20%), as the R(140) feature is not listed in HITRAN. For the R(103) and R(104) features used to measure $T_{\text {vib}}$, the relative error from each term in Eq. 17 is shown in Fig. 14. The two primary sources of error are the uncertainty in rotational temperature and uncertainty in absorbance area fit. As this analysis shows, uncertainty in $T_{\text {vib}}$ is strongly correlated to the $T_{\text {rot}}$ uncertainty and could be further reduced if needed by improving the $T_{\text {rot}}$ accuracy.

Rotational temperature uncertainty can be estimated via Eq. 19 (see Minesi et al. [35]), provided the spectral features are within the same vibrational band.

19 $$\begin{aligned} {\frac{\delta T}{T} = \frac{k_B}{hc}\frac{T}{\Delta E}\sqrt{\sum _{i = 1}^2\left( \frac{\delta S_i}{S_i}\right) ^2 + \left( \frac{\delta A_i}{A_i}\right) ^2 } }. \end{aligned}$$

Considering the fundamental $\nu _3$(01$^1$0) R(103) and R(140) features used in this work, the estimated uncertainty is 13% assuming the HITEMP value of 20% for $\delta$S/S of R(140). Based on the consistency of our measurements validated via normal shock relations, we estimate $\delta$S/S to be 10% for the R(140) feature, thus yielding a more realistic uncertainty of 8% for $\delta$T$_{\text {rot}}$/T$_{\text {rot}}$.

### Non-equilibrium $T_{\text {vib}}$ sensitivity

Vibrational temperature is determined from the absolute area measurement of the R(103) and R(104) spectral features in the 01$^1$0 vibrational lower state in this work. It is hypothesized that non-equilibrium between vibrational modes may exist at certain conditions in this study. Sensitivity of the area measurement was analyzed with respect to different vibrational temperatures.

20 $$\begin{aligned}S_{j}& = \frac{A_{21}g_{2}}{8\pi \nu ^2cQ_\text {rot}(T_\text {rot})Q_\text {vib}(T_\text {vib,ss},T_\text {vib,bend}, T_\text {vib,as})} \nonumber \\&\quad \biggl [\exp \left( \frac{-c_2E_{\text {rot},1}}{T_\text {rot}}\right) \exp \left( \frac{-c_2E_{\text {vib,bend,1}}}{T_\text {vib,bend}}\right) \nonumber \\&\quad -\exp \left( \frac{-c_2E_{\text {rot},2}}{T_\text {rot}}\right) \exp \left( \frac{-c_2E_{\text {vib,bend,2}}}{T_\text {vib,bend}}\right) \exp \left( \frac{-c_2E_{\text {vib,as,2}}}{T_\text {vib,as}}\right) \biggr ]. \end{aligned}$$

The analysis requires the $T_{\text {vib}}$ and $E_{\text {vib}}$ terms in Eq. 8 to be separated into three separate terms with an associated temperature for each mode: symmetric stretch - $T_{\text {vib,ss}}$, bending - $T_{\text {vib,bend}}$, and asymmetric stretch - $T_{\text {vib,as}}$. The linestrengh expression then becomes Eq. 20 and the non-equilibrium partition function can be calculated using the Klarenaar model [15]. To simplify the calculation, we assume $T_{\text {vib,ss}}$ = $T_{\text {vib,bend}}$, as this is often assumed due to Fermi resonance between these modes. The sensitivity to each vibrational temperature can be calculated by taking the respective temperature derivative of each mode. This is done numerically and shown in Fig. 15 at T$_{\text {tr-rot}}$ = 2250 K. The sensitivity of each vibrational mode temperature is not a strong function of rotational temperature.

It is observed that the dominant signal in the area of the R(103) (and similarly R(104)) transition is the bending mode temperature over the entire range investigated in this study (T = 1250–3000 K). The R(103) area is approximately twice as sensitive to the bending mode temperature when compared with the asymmetric stretch temperature. This is expected as $T_{\text {vib,as}}$ effects the stimulated emission of the resolved areas, and its influence grows with increasing T$_{\text {vib,as}}$. In conclusion, this analysis shows the relative influence of potential non-equilibrium across the vibrational modes on the state populations inferred from the R(103) and R(104) lines. At early test times, if $T_{\text {vib,bend}}>T_{\text {vib,as}}$, the sensor can be interpreted as resolving mostly the bending mode temperature with a high degree of confidence considering the curves in Fig. 15.
